# Association of clonal hematopoiesis of indeterminate potential with myocardial characteristic differences in non-ischemic heart failure

**DOI:** 10.1016/j.heliyon.2025.e42858

**Published:** 2025-02-20

**Authors:** Jooyeon Lee, Yoo Jin Hong, Jin-Ho Park, Su-Yeon Choi, Chansub Lee, Choonghyun Sun, Hongyul An, Youngil Koh, Se-Eun Kim, Jaewon Oh, Seok-Min Kang, Chan Joo Lee, Young-Jin Kim

**Affiliations:** a Division of Cardiology, Department of Internal Medicine, Severance Hospital, Yonsei University College of Medicine, Republic of Korea; b Department of Radiology, Research Institute of Radiological Science, Severance Hospital, Yonsei University College of Medicine, Republic of Korea; c Department of Family Medicine, Seoul National University Hospital and Seoul National University College of Medicine, Seoul, Republic of Korea; d Department of Internal Medicine, Seoul National University College of Medicine, Seoul National University Hospital Healthcare System Gangnam Center, Seoul, Republic of Korea; e Genome Opinion Incorporation, Seoul, Republic of Korea; f Department of Internal Medicine, Seoul National University Hospital, Seoul, Republic of Korea

**Keywords:** Somatic mutation, Non-ischemic cardiomyopathy, Heart failure, Cardiac magnetic resonance imaging

## Abstract

Clonal hematopoiesis of indeterminate potential (CHIP) is an aging process associated with the prognosis in heart failure (HF), regardless of etiology. This study investigated the association between CHIP and myocardial tissue characteristics in patients with non-ischemic HF. We enrolled 95 patients diagnosed with non-ischemic HF with a left ventricular ejection fraction (LVEF) of ≤40 % who underwent cardiac magnetic resonance imaging (MRI) within 3 months of diagnosis. Next-generation sequencing was performed to determine the presence of CHIP-related mutations. Transthoracic echocardiography was performed to evaluate left ventricular reverse remodeling. CHIP was identified in 15 patients. Patients with CHIP-mutation had higher native T1, extracellular volume fraction, and late gadolinium enhancement quantification (LGE). After adjusting for age and sex, CHIP mutations remained as contributing factors to the differences in cardiac MRI mapping values. However, no difference was observed in left ventricular reverse remodeling by CHIP within 1 year. This study demonstrated CHIP’s association with higher mapping values and LGE on cardiac MRI. However, its impact on short-term LV reverse remodeling in non-ischemic HF patients was attenuated, indicating the need for further research on its long-term effect.

## Graphical abstract

In non-ischemic HFrEF, patients with CHIP mutations, such as native T1, ECV, and T2, have higher mapping values on cardiac MRI performed during diagnosis than those without CHIP mutations.CHIP, clonal hematopoiesis of indeterminate potential; ECV, extracellular volume; HFrEF, heart failure with reduced ejection fraction.

**Figure:**
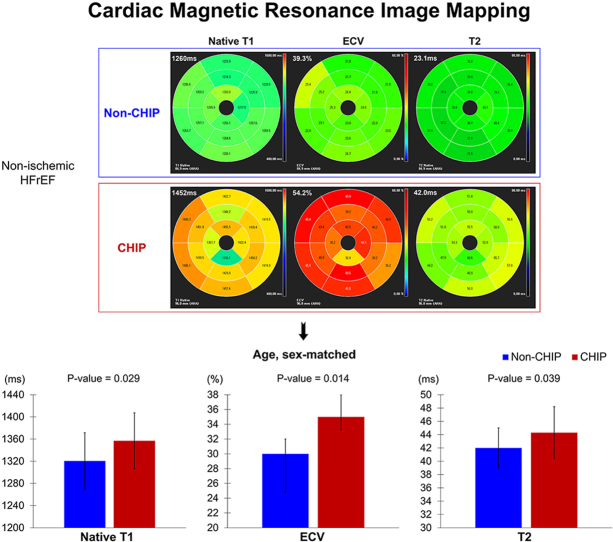

## 1 Introduction

Clonal hematopoiesis (CH) is the clonal expansion of hematopoietic cells that carry somatic mutations, resulting in the circulation of mutant blood cells in peripheral blood [1]. When these mutations occur without evidence of hematologic malignancy, the condition is referred to as clonal hematopoiesis of indeterminate potential (CHIP) [2]. The prevalence of CHIP increases with age, detected in over 5 % of individuals in their 60s and more than 10 % in their 70s, while it is rare in individuals under 40 years of age [3]. This suggests that CHIP is commonly observed in aging individuals and may be a contributor to age-related diseases.

Heart failure (HF), which is strongly linked to aging, affects approximately 1 in 10 individuals over the age of 80. Of these, 60 % are attributed to ischemic etiology, while the remaining 40 % are classified as non-ischemic [4,5]. Recent studies have indicated that CHIP may be linked to an increased risk of cardiovascular disease (CVD), particularly ischemic CVD, as well as early-onset myocardial infarction, mortality, and rehospitalization [1], suggesting that CHIP promotes a pro-inflammatory state that accelerates disease progression [6]. However, emerging evidence also points to CHIP’s prognostic significance in heart failure of non-ischemic etiology, though the underlying mechanisms remain unclear [3,7]. Myocardial tissue characteristics, as in myocardial fibrosis and inflammation, are well-established prognostic factors in non-ischemic HF [8]. Persistent low-grade inflammation promotes myofibroblast formation, interstitial collagen deposition, reactive oxygen species production, and reduces nitric oxide bioavailability, all of which contribute to the development of HF and are associated with a poor prognosis [9]. Despite these known associations, the underlying mechanisms driving these tissue changes, particularly in the absence of atherosclerosis, remain poorly understood. Therefore, this study aims to investigate the association between CHIP and myocardial tissue characteristics in patients with non-ischemic HF to better understand the potential role of CHIP in disease progression in this patient population.

## 2 Results

### 2.1 Prevalence of CHIP mutations

**Fig. 1:**
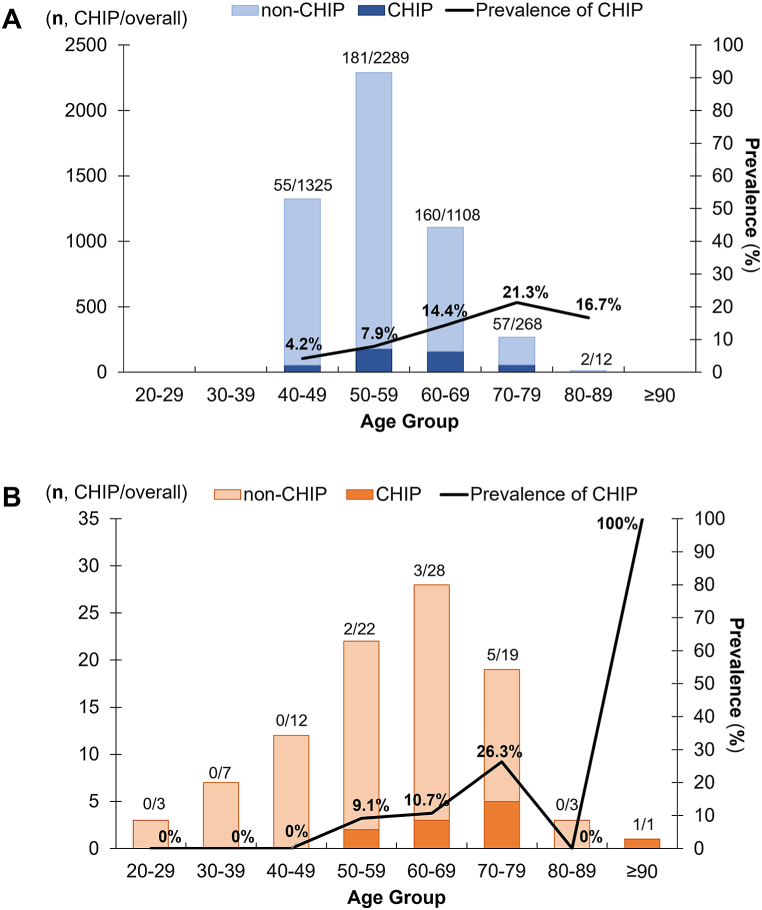
Prevalence of CHIP mutation with variant allelic fraction (VAF) ≥1.5 in GENIE cohort and non-ischemic HFrEF patients (CH, clonal hematopoiesis of indeterminate potential; GENIE, cohort study of individuals who underwent a routine health checkup; HFrEF, heart failure with reduced ejection fraction).

The prevalence of CHIP mutations appeared to be higher, though not statistically significant, in the non-ischemic HFrEF group compared to the GENIE cohort, in which ones who underwent a routine health checkup and serve as a healthy control group. All CHIP mutations were identified in patients over 50-year-old in the non-ischemic HFrEF group, with prevalence increasing with age, aligning with the clinical characteristics observed in the general population (Fig. 1). With a VAF criterion of ≥1.5 %, the prevalence of CHIP was approximately 15.8 % (n = 15) and 11.6 % (n = 11) with a VAF criterion of ≥2 %. In comparison, the prevalence was 12.0 % and 9.6 % in the GENIE cohort (Fig. 1 and Table S1). The detected CHIP-related gene variants in non-ischemic HFrEF according to VAF are depicted in Fig. S1. Furthermore, the prevalence of *TET2* mutations was higher in the non-ischemic HFrEF group than in the GENIE cohort (*p* = 0.037), and this difference remained statistically significant after being analyzed with a logistic regression model adjusted for age and sex. (Table S2).

### 2.2 Baseline characteristics according to CHIP mutations

A total of 95 patients with non-ischemic HF were analyzed, with 25.5 % of patients over 50 years old. Among them, 15.8 % (n = 15) have CHIP mutations and were older than those without mutation (67.6 years vs. 57.4 years, *p* = 0.009). The distribution of clinical risk factors, heart rhythm, baseline blood pressure, and heart rate did not differ significantly according to CHIP mutations. However, in laboratory findings, patients with CHIP mutations tended to be more anemic (12.8 g/dL vs. 13.9 g/dL, *p* = 0.060) and elevated NT-proBNP levels (1105.0 pg/mL vs. 175.5 pg/mL, *p* = 0.018). Nonetheless, these differences were mitigated in the age- and sex-matched analyses (Table S3). Moreover, medications used at outpatient clinics did not differ based on CHIP status, with more than 80 % of patients receiving guideline-directed medical therapy (GDMT) for HF (Table S4). Additionally, there was no occurrence of valvular heart disease in patients with CHIP mutations (Table 1).

**Table 1:** Baseline characteristics of patients by CHIP mutations.

	CHIP (n = 15)	Non-CHIP (n = 80)	*p*-value
***Demographics***			
Age, years	67.6 ± 9.6	57.4 ± 14.2	**0.009**
Male, n (%)	10 (66.7 %)	53 (66.2 %)	1.000
Hypertension, n (%)	7 (46.7 %)	31 (38.8 %)	0.774
Diabetes, n (%)	6 (40.0 %)	19 (23.8 %)	0.321
CAOD, n (%)	1 (6.7 %)	5 (6.2 %)	1.000
Previous stroke, n (%)	1 (6.7 %)	3 (3.8 %)	1.000
VHD, n (%)	0 (0 %)	5 (6.2 %)	0.549
SBP, mmHg	123.5 ± 28.1	128.6 ± 20.8	0.416
DBP, mmHg	78.9 ± 16.8	84.7 ± 17.3	0.232
HR, bpm	90.0 ± 25.9	88.4 ± 24.8	0.820
Heart rhythm, n (%)			0.903
Atrial fibrillation	3 (20.0 %)	20 (25.0 %)	
Atrial flutter	1 (6.7 %)	6 (7.5 %)	
Sinus	11 (73.3 %)	54 (67.5 %)	
LBBB, n (%)	2 (13.3 %)	14 (17.5 %)	0.984
***Laboratory findings***			
Hb, g/dL	12.8 [12.2; 14.0]	13.9 [12.9; 14.9]	0.060
Total protein, g/dL	7.0 ± 0.6	7.2 ± 0.5	0.285
Albumin, g/dL	4.3 ± 0.6	4.5 ± 0.3	0.237
Glucose, mg/dL	121.2 ± 29.3	111.7 ± 35.1	0.377
Total cholesterol, mg/dL	146.0 ± 38.6	157.3 ± 39.6	0.371
Triglyceride, mg/dL	112.8 ± 51.5	136.1 ± 91.7	0.487
HDL-cholesterol, mg/dL	43.6 ± 6.8	47.0 ± 11.0	0.401
LDL-cholesterol, mg/dL	70.2 ± 13.4	97.1 ± 40.8	0.208
BUN, mg/dL	21.9 ± 9.1	19.8 ± 7.5	0.340
Cr, mg/dL	1.7 ± 2.3	1.0 ± 0.3	0.233
Na, mmol/L	138.5 ± 3.2	139.2 ± 2.7	0.367
K, mmol/L	4.5 ± 0.4	4.5 ± 0.5	0.792
NT-proBNP, pg/mL	1105.0 [271.0; 1695.0]	175.5 [94.0; 540.0]	**0.018**

CAOD, coronary artery occlusive disease; LBBB, left bundle branch block; SBP, systolic blood pressure; DBP, diastolic blood pressure; HR, heart rate; Hb, hemoglobin; HDL-cholesterol, high-density lipoprotein-cholesterol; LDL-cholesterol, low-density lipoprotein-cholesterol; NT-proBNP, N-terminal pro b-type natriuretic peptide; VHD, valvular heart disease.

### 2.3 Cardiac MRI parameters by CHIP status

**Fig. 2:**
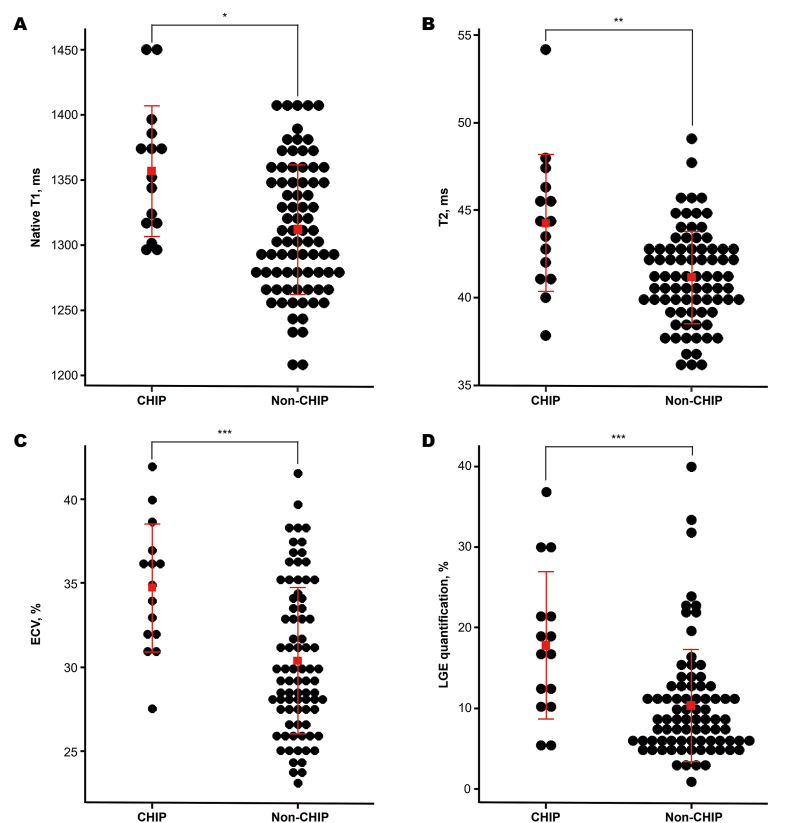
Distribution of MRI mapping for native T1 (A), T2 (B), ECV (C) and LGE quantification (D) in age, sex-matched subjects according to CHIP mutations (CHIP, clonal hematopoiesis of indeterminate potential; ECV, extracellular volume; LGE, late gadolinium enhancement. ∗*p*-value: 0.002, ∗∗*p*-value: 0.010, ∗∗∗*p*-value: 0.001).

Using cardiac MRI, the assessed LVEF was comparable across the groups (26.6 % vs. 27.5, *p* > 0.05), as well as LV stroke volume (58.5 mL vs. 59.8 mL) and cardiac output (4.1 L/min vs. 4.4 L/min). However, the MRI mapping analysis presented the mean native T1 value (1356.8 ms vs. 1312.2 ms, *p* = 0.002), median ECV (35.0 % vs. 29.4 %, *p* = 0.001), and LGE quantification (16.9 % vs. 8.5 %, *p* = 0.001) were heightened in CHIP mutation group compared to the non-CHIP group, even after matching for age and sex. These results remained robust in multivariate regression analysis with adjusted age and sex. Additionally, the mean T2 value was higher in individuals with CHIP mutations (44.3 ms vs. 41.2 ms, *p* = 0.010), as confirmed by age- and sex-matched analyses. However, the multivariable-adjusted analyses did not reveal statistically significant associations of T2 value with CHIP (refer to Table 2, Table 3, Fig. 2).

**Table 2:** Cardiac MRI findings according to CHIP mutation.

	Total population			Age, sex-matched		
	CHIP (n = 15)	Non-CHIP (n = 80)	*p*-value	CHIP (n = 15	Non-CHIP (n = 30)	*p*-value
LVEDV, ml	239.9 ± 76.9	233.3 ± 80.6	0.770	239.9 ± 76.9	200.2 ± 74.4	0.103
LVESV, ml	181.4 ± 71.0	173.5 ± 77.4	0.715	181.4 ± 71.0	147.1 ± 69.7	0.130
LVSV, ml	58.5 ± 20.4	59.8 ± 21.0	0.827	58.5 ± 20.4	53.1 ± 22.5	0.436
LVEF, %	26.6 ± 11.4	27.5 ± 9.9	0.748	26.6 ± 11.4	28.1 ± 10.1	0.645
LVCO, l/min	4.1 ± 1.4	4.4 ± 1.5	0.485	4.1 ± 1.4	3.9 ± 1.4	0.591
LVCI, l/min/m^2^	2.4 ± 0.8	2.4 ± 0.7	0.946	2.4 ± 0.8	2.3 ± 0.7	0.679
RVEDV, ml	161.1 ± 62.9	176.1 ± 65.1	0.413	161.1 ± 62.9	145.9 ± 59.9	0.435
RVESV, ml	105.5 ± 58.6	117.4 ± 59.9	0.482	105.5 ± 58.6	93.3 ± 48.3	0.460
RVSV, ml	55.6 ± 17.6	58.7 ± 20.8	0.587	55.6 ± 17.6	52.6 ± 23.3	0.668
RVEF, %	37.2 ± 12.9	35.8 ± 12.6	0.708	37.2 ± 12.9	37.7 ± 12.6	0.898
RVCO, l/min	3.9 ± 1.3	4.3 ± 1.5	0.326	3.9 ± 1.3	3.8 ± 1.5	0.829
RVCI, l/min/m^2^	2.3 ± 0.7	2.4 ± 0.7	0.686	2.3 ± 0.7	2.3 ± 0.8	0.947
Native T1, ms	1356.8 ± 50.4	1312.2 ± 50.2	**0.002**	1356.8 ± 50.4	1320.6 ± 50.8	**0.029**
T2, ms	44.3 ± 3.9	41.2 ± 2.7	**0.010**	44.3 ± 3.9	42.0 ± 3.0	**0.039**
ECV, %	35.0 [32.0; 36.7]	29.4 [27.3; 33.8]	**0.001**	35.0 [32.0; 36.7]	30.0 [28.0; 35.2]	**0.014**
LGE quantification, %	16.9 [11.4; 21.5]	8.5 [5.8; 12.5]	**0.001**	16.9 [11.4; 21.5]	8.2 [5.4; 12.8]	**0.002**

CI, cardiac index; CO, cardiac output; ECV, extracellular volume; EDV, end-diastolic volume; EF, ejection fraction; ESV, end-systolic volume; LGE, late gadolinium enhancement; LV, left ventricle; RV, right ventricle

**Table 3:** Multivariate regression model of factors associated with MRI mapping and CHIP mutations.

(A) Total population								
	Native T1		T2		ECV		LGE	
	*β* (SE)	*p*-value	*β* (SE)	*p*-value	*β* (SE)	*p*-value	*β* (SE)	*p*-value
Age	0.10 (0.39)	0.802	0.06 (0.02)	**0.006**	0.04 (0.03)	0.210	0.01 (0.06)	0.979
Male	−23.76 (10.99)	**0.033**	−0.41 (0.62)	0.511	−0.83 (0.96)	0.386	1.39 (1.63)	0.398
EF	−0.76 (0.67)	0.261	−0.02 (0.04)	0.563	0.01 (0.06)	0.870	0.16 (0.10)	0.106
CHIP	41.96 (14.68)	**0.005**	2.40 (0.83)	**0.004**	3.93 (1.28)	**0.003**	7.80 (2.18)	**0.000**

(B) In age, sex-matched subjects by CHIP mutations								
	Native T1		T2		ECV		LGE	
	*β* (SE)	*p*-value	*β* (SE)	*p*-value	*β* (SE)	*p*-value	*β* (SE)	*p*-value
Age	0.39 (0.87)	0.657	0.06 (0.06)	0.261	0.09 (0.07)	0.250	−0.03 (0.12)	0.821
Male	−27.65 (16.18)	**0.010**	−0.10 (1.06)	0.925	−0.70 (1.39)	0.615	1.47 (2.18)	0.503
EF	−0.32 (0.97)	0.741	−0.08 (0.06)	0.228	−0.03 (0.08)	0.715	0.20 (0.13)	0.130
CHIP	40.18 (16.66)	**0.021**	1.88 (1.09)	0.093	3.46 (1.43)	**0.020**	8.70 (2.25)	**0.000**

CHIP, Clonal Hematopoiesis of Indeterminate Potential; ECV, extracellular volume; EF, ejection fraction; LGE, late gadolinium enhancement; SE, standard error

### 2.4 Changes in echocardiographic parameters and left ventricular reverse remodeling

The changes in echocardiographic parameters are illustrated in Table 4. At baseline, the LVEF (26.7 % vs. 28.9 %) and LV cardiac chamber sizes (LVEDD: 62.5 mm vs. 63.1 mm) did not differ statistically, nor did the degree of improvement in cardiac function during the follow-up period, stratified by CHIP mutation status. Patients were followed up for a median of 180 days (IQR, 109–248 days). However, CHIP mutations did not predict left ventricular reverse remodeling (LVRR) or the incidence of LVRR at follow-up for HFrEF, even after age- and sex-matched analyses.

**Table 4:** Changes in echocardiographic parameters and associated left ventricular reverse remodeling in the age, sex-matched subjects according to CHIP mutations.

	Total population			age, sex-matched subjects		
	CHIP (n = 15)	Non-CHIP (n = 80)	*p*-value	CHIP (n = 15)	Non-CHIP (n = 30)	*p*-value
*Baseline TTE*						
LVEF, %	26.7 ± 8.6	28.9 ± 7.9	0.311	26.7 ± 8.6	31.0 ± 8.2	0.110
LVEDD, mm	62.5 ± 10.2	63.1 ± 8.0	0.810	62.5 ± 10.2	60.8 ± 8.4	0.539
LVESD, mm	54.0 ± 10.4	54.4 ± 8.4	0.883	54.0 ± 10.4	51.6 ± 8.7	0.423
LVMI, g/m^2^	153.0 ± 49.3	147.9 ± 40.9	0.667	153.0 ± 49.3	153.5 ± 52.2	0.977
LAVI, g/m^2^	52.1 ± 23.4	53.8 ± 21.9	0.790	52.1 ± 23.4	60.2 ± 26.9	0.328
E velocity, m/s	0.9 ± 0.3	0.7 ± 0.3	0.202	0.9 ± 0.3	0.7 ± 0.2	0.179
E′ velocity, m/s	4.8 ± 2.3	4.8 ± 2.0	0.981	4.8 ± 2.3	4.4 ± 2.1	0.602
E/E′	20.7 ± 10.9	17.4 ± 9.3	0.250	20.7 ± 10.9	17.8 ± 7.1	0.333
*3-6 month follow-up TTE*						
LVEF, %	37.3 ± 14.6	43.6 ± 14.0	0.116	37.3 ± 14.6	45.2 ± 14.7	0.100
LVEDD, mm	59.3 ± 10.1	57.3 ± 7.1	0.356	59.3 ± 10.1	55.1 ± 8.3	0.149
LVESD, mm	48.7 ± 12.0	44.4 ± 9.0	0.107	48.7 ± 12.0	42.6 ± 10.7	0.089
LVMI, g/m^2^	144.0 ± 49.2	123.3 ± 25.3	0.131	144.0 ± 49.2	120.6 ± 29.6	0.106
LAVI, g/m^2^	48.7 ± 22.3	39.2 ± 20.6	0.109	48.7 ± 22.3	45.0 ± 26.0	0.640
E velocity, m/s	0.7 ± 0.3	0.6 ± 0.2	0.245	0.7 ± 0.3	0.6 ± 0.2	0.202
E′ velocity, m/s	5.0 ± 2.6	5.2 ± 1.7	0.785	5.0 ± 2.6	4.6 ± 1.4	0.601
E/E′	15.0 ± 8.1	11.9 ± 4.5	0.172	15.0 ± 8.1	12.8 ± 3.8	0.328
*Changes in TTE in 3-6 month follow-up*						
Change in EF, %	10.7 ± 13.4	14.7 ± 13.1	0.278	10.7 ± 13.4	14.0 ± 13.0	0.435
Improved EF, n (%)	11 (73.3 %)	71 (91.0 %)	0.132	11 (73.3 %)	26 (89.7 %)	0.333
HFimpEF at f/u, n (%)	5 (33.3 %)	35 (44.9 %)	0.588	5 (33.3 %)	13 (44.8 %)	0.681

LVEDD, left ventricular end-diastolic diameter; LVESD, left ventricular end-systolic diameter; LV, left ventricle; EF, ejection fraction; LVMI, left ventricular mass index; LAVI, left atrium volume index.

## 3 Discussion

In this study, individuals with CHIP mutations in the general population were consistently aged 50 or older, while the prevalence of CHIP mutations in non-ischemic HF appeared to be higher than in the general population. Since CHIP-related genes are associated with aging, the observed results may be influenced by age differences between the non-ischemic HFrEF group and the GENIE cohort (59 years vs. 55 years, *P* = 0.009). However, *TET2* mutation appears to be more prevalent among individuals with non-ischemic Among non-ischemic HF patients, apart from those with CHIP mutations who were elder, there are no significant differences in clinical characteristics or echocardiographic parameters, including LV chamber size and ejection fraction, across the groups. Nevertheless, the change in myocardial tissue characteristics of non-ischemic HF patients with CHIP mutation, as evidenced by elevated native T1, T2, ECV and quantified LGE values in the cardiac MRI, suggests a potential association with the prognosis of non-ischemic heart failure. These findings provide a plausible explanation for CHIP as a risk factor for the poor long-term prognosis of HF, as indicated in previous research. (refer to Graphic Abstract). While the short-term outcomes of LV reverse remodeling were not significantly impacted, the myocardial tissue changes observed in patients with CHIP mutations, especially the elevated T1, T2, and ECV values, suggest that CHIP mutations may contribute to a worse long-term clinical course. This has implications for early intervention and closer monitoring in clinical practice.

CHIP, commonly involving the genes variants involved in epigenetic regulation, such as *ASXL1*, *TET2*, and *JAK2,* is an independent risk factor for HF regardless of etiology [3,10]. Moreover, evidence suggests CHIP-related mutations lead to “inflammaging”, which is associated with the progression of aging-related diseases, including atherosclerotic CVD [1] and HF [10]. Additionally, these inflammatory responses involved several immune cells infiltrating the myocardium and promoting cardiac remodeling [11]. The inflammatory response in the heart aims to resolve myocardial damage, promptly adapt to abnormal conditions and restore homeostasis and cardiac function [12]. However, persistent inflammation and circulation of blood cytokines from systemic inflammatory diseases impair myocardial contractility [9,13], leading to progressive left ventricular remodeling and deterioration, contributing to the progression and exacerbation of HF [14]. These findings suggest that the inflammatory and fibrotic changes associated with CHIP mutations may be early indicators of poor long-term prognosis, with potential effects on survival and hospitalization rates, even though the short-term outcomes in LV reverse remodeling were not affected.

Among the CHIP subtypes, our study observed a higher prevalence of *TET2* (Tet methylcytosine dioxygenase 2 gene, encoding proteins of epigenetic regulatory enzyme) mutation in patients with non-ischemic HFrEF compared to the general population. In animal models, the *TET2* deficiency leads to exacerbated IL-1β production in the maladaptive cardiac remodeling [15] and accelerated age-related cardiomyopathy with contractile dysfunction, LV hypertrophy, and exacerbated cardiac fibrosis without deleterious stimuli [16].

Surprisingly, the incidence of CHIP between patients with non-ischemic HF and the general population was comparable in this study, leading to speculate the presence of CHIP may not be a powerful contributor to the heightened risk of developing non-ischemic HFrEF. However, the inference regarding the more severe myocardial injury observed in patients with non-ischemic HFrEF with CHIP versus without mutations was confirmed using cardiac MRI [6]. Cardiac MRI is a non-invasive imaging modality highly capable of characterizing myocardial tissue and is an essential test for diagnosing and following non-ischemic HFrEF, predicting prognosis and assessing treatment response. In the heart, microvascular endothelial inflammation promotes the expression of adhesion molecules that attract circulating leukocytes, forming myofibroblasts and interstitial collagen deposition [9]. T2 mapping abnormalities, reflected by prolongation of T2 relaxation time, generally correspond to intra-cardiomyocyte edema on cardiac MRI, even in the presence of normal T1 and ECV [17]. Meanwhile, LGE and ECV values on cardiac MRI are associated with interstitial and replacement myocardial fibrosis [18]. Our results showed an overall increase in T2 values, especially in patients with CHIP-related mutations, suggesting that myocardial edema is present in non-ischemic HFrEF in a relatively acute or subacute setting, and there may have more severe myocardial edema in response to injury. These findings support the hypothesis that CHIP mutations contribute to ongoing myocardial inflammation and edema, which, over time, may lead to the worsening of heart failure and associated long-term adverse outcomes.

In addition, higher quantified LGE, ECV, and T1 values were shown, which may indicate that CHIP contributes to severe interstitial and myocardial inflammation dysregulation with replacement fibrosis under pressure or volume overload in non-ischemic HFrEF [18]. Studies suggested *TET2* and DNA methyltransferase 3 alpha (*DNMT3A*) mutation, the most common genetic mutation in CHIP, accelerate the progression of HFrEF irrespective of etiology [3]. Furthermore, *TET2* CHIP was found to be an independent risk factor associated with HF beyond reduced ejection fraction (EF ≥ 50 %), primarily reflecting the association with new-onset HF [19]. In animal models, the inactivation of *DNMT3A* in macrophages facilitates the activation of cardiac fibroblasts and increases cardiac fibrosis [20], which aligned with our study findings and have translated the evidences from prior animal studies into clinical insights in patients with non-ischemic HFrEF.

Besides, LV reverse remodeling indicates a decrease in end-diastolic volume and an increase in LVEF in patients with HFrEF and has a positive impact on the prognosis of HFrEF [21]. It occurred in approximately one-third of the patients with new-onset non-ischemic HFrEF after GDMT optimization [[22], [23], [24]]. In our study, LV reverse remodeling occurred within 3–6 months in approximately 40 % of the patients. Although CHIP mutations were associated with worse myocardial characteristics on MRI, indicating a poor substrate of the myocardium, they were not associated with less LV reverse remodeling. This can be attributed to the short follow-up duration (median 180 days), even though optimal GDMT was delivered regardless of CHIP mutation in non-ischemic HFrEF patients, where LV reverse remodeling can be observed for up to 2 years, requiring a longer follow-up [21]. Nonetheless, although CHIP does not negatively affect short-term LV reverse remodeling, the negative changes in myocardial tissue with increased native T1, ECV, and T2 values seen in patients with CHIP mutations confirmed by cardiac MRI, may partially explain the impact of CHIP on the clinical course of HF, particularly in the long term, where persistent myocardial injury could lead to disease progression.

The present study is the first observational study to examine the association between CHIP and structural and functional aspects of the heart, utilizing cardiac MRI to obtain information on myocardial tissue characteristics and assess early LV reverse remodeling under GDMT. Nevertheless, larger prospective studies are warranted with a prolonged follow-up duration of at least one to two years and additional follow-up cardiac MRI to further explore changes in the inflammatory and fibrosis phases of the myocardial issue and identify sentinel risk factors in the progression to the early phase of non-ischemic HF. Longer follow-up is essential to validate whether CHIP mutations contribute to the worsening of heart failure over time, and to better define their role as a prognostic marker in long-term clinical outcomes.

This study presents the association of CHIP-related mutations with the changes in myocardial tissue characteristics resulting in elevated native T1, T2, and ECV values and quantified LGE, as observed in cardiac MRI. While short-term improvement in cardiac function, particularly in LV reverse remodeling, was not evident, the pro-inflammatory effect of CHIP contributing to increased myocardial edema and fibrosis implies potential long-term implications in non-ischemic HF, which merits validations in future prospective studies.

## 4 Limitations of the study

This study has several limitations. First, the small sample size and short duration of follow-up limit the assessment of long-term implications. Second, compared to the prevalence of CHIP observed in the previous study, lower prevalence of CHIP (39 % vs. 15.8 %) was identified [3]. This discrepancy might be attributable to the inclusion of more than 30 % of the patients with ischemic etiology older than our study population. Third, performing CHIP-related gene sequencing after cardiac MRI introduces temporal biases, raising uncertainty about whether the presence of CHIP is associated with HF and changes in CHIP status over time. Fourth, limited imaging modalities, such as positron emission tomography scans or histological analysis, were used in addition to cardiac MRI. Lastly, the uniformity in the etiology of HF within the study population limited the focus on CHIP mutations to non-ischemic HF, which introduces inherent design limitations. Differentiating ischemic from non-ischemic HF would enhance clinical relevance and offer deeper insights into the specific role of CHIP in non-ischemic HF. Nonetheless, these limitations will likely have minimal effect on our study findings, as they were consistent in sensitivity analyses incorporating the standard of CHIP to be VAF ≥2 %, confirming the association between CHIP and myocardial tissue characteristics in non-ischemic HFrEF (Tables S5–S7).

## 5 Methods

### 5.1 Study participants

This study enrolled patients with HF with reduced ejection fraction (HFrEF) treated in an outpatient setting from November 2021 to January 2023. The inclusion criteria were as follows: 1) Patients ≥19 years of age; 2) a history of left ventricular ejection fraction (LVEF) of <40 % on imaging tests within 3 years (HFrEF diagnosis) in patient; 3) patients who underwent cardiac magnetic resonance imaging (CMR) taken within 3 months of being diagnosed with HFrEF; and 4) patients diagnosed with non-ischemic HF by performing coronary artery imaging (angiography, computed tomography [CT]) at the time of HFrEF diagnosis. Exclusion criteria were as follows: 1) confirmed ischemic cardiomyopathy (stenosis of ≥75 % of the major coronary arteries confirmed on coronary artery imaging or ischemic cardiomyopathy findings, such as subendocardial or transmural late gadolinium enhancement on CMR); 2) history of solid cancer diagnosis and chemotherapy; and 3) history of hematologic malignancy. All laboratory and clinical information was collected from electronic medical records during HF diagnosis. These patients received guideline-directed medical treatment for HFrEF. Blood sampling was conducted after enrollment in this study to confirm the presence of CHIP mutations.

To compare the prevalence of CHIP-related mutations in patients with non-ischemic HF to that in the general population, data from the Gene-Environment of Interaction and Phenotype (GENIE) cohort were utilized. The GENIE cohort has been collecting blood prospectively since 2013 from the Seoul National University Hospital Healthcare System Gangnam Center, which conducts comprehensive Korean health examinations. A total of 5486 participants from the GENIE cohort were tested for CHIP mutations. The details of the cohort have been previously described [25].

### 5.2 Ethical approval

This study was conducted in accordance with the principles of the Declaration of Helsinki. The study protocol was reviewed and approved by the Institutional Review Board (IRB) of the Severance hospital (IRB number: 4-2021-0921; clinical trials. gov identifier: NCT05981144) and of the Seoul National University Hospital (IRB number: H-1908-121-1056 and: H-2201-121-1295). Written informed consent was obtained from all patients before their inclusion.

### 5.3 Transthoracic echocardiography analysis and follow-up

Echocardiographic parameters were collected at the time of HF diagnosis and 3–6 months after diagnosis. Left ventricular (LV) systolic function was estimated using the biplane method, and the LV end-diastolic dimension (LVEDD) was measured using M-mode tracing or 2D-guided linear measurement. LV reverse remodeling (LVRR) was determined in accordance with the criteria of HF with improved ejection fraction (EF) in the 2022 European Society of Cardiology HF guidelines and was defined to meet both of the following criteria: 1) ≥10 % absolute improvement in LVEF; and 2) follow-up LVEF >40 % [26,27].

### 5.4 Sample collection and DNA analysis

On the same day, patients with HFrEF consented to the study, 10 ccs of peripheral blood were collected in EDTA tubes, refrigerated, and centrifuged at 2000 g at 4 °C within 24 h of collection. Peripheral blood mononuclear cells (PBMCs) were separated from whole blood and stored at −20 °C until DNA extraction. Then, DNA was extracted from PBMCs using a Geneall Exgene blood SV mini prep kit (Geneall/Cat. No.105-152). NGS library was prepared using Twist Library Preparation EF Kit. A targeted next-generation sequencing technique was then applied to a specifically designed set of 89 genes often associated with CHIP, including key genes such as *DNMT3A, TET2, ASXL1, JAK2*, and *TP53*. This was performed at a high precision level, ensuring the sequencing depth was over 1000 times. A variant allele frequency (VAF) from 1.5 % to 30 % of the DNA isolated from peripheral blood was identified as a potential CHIP-related mutation. To ensure specificity, common genetic variations that are widely present in the general population, as documented in extensive genetic databases, such as gnomAD, 1000 Genomes Project version 3, ESP6500, and ExAC, as well as those found in a reference group of 1000 Koreans, were omitted from consideration.

### 5.5 Cardiac MRI analysis

Cardiac magnetic resonance imaging (MRI) was performed using a 3-T MR scanner (3T, Prisma fit, Siemens Healthineers) equipped with a 30-channel array body coil. Cardiac MRI included cardiac cine, native T1 and T2, post-T1 mapping, and late gadolinium enhancement (LGE). Cine images were obtained in the 4- and 2-chamber views, capturing one plane and short-axis cine images covering the entire bilateral ventricle. A retrospective electrocardiography gating technique used a balanced steady-state free-precession sequence (true fast imaging with steady-state precession [TrueFISP]). Native T1 mapping images were acquired using a modified look-locker inversion-recovery 5(3)3 (MOLLI) sequence in three short-axis planes (apical, mid, and base of the LV) by applying a nonselective inversion pulse (TrueFISP single-shot readout sequence) in the mid-diastolic phase) with the following parameters: field of view, 306 × 360 mm; acquisition matrix, 144 × 256; slice thickness, 8 mm; TR, 2.24 ms; TE, 1.12 ms; minimum inversion time, 100 ms; inversion time increment, 80 ms; flip angle, 35°; parallel acquisition acceleration factor, 2; number of inversions, 3. Five images were acquired after the first inversion, and following a pause or three heartbeats, three images were acquired after the second inversion. Fully automated nonrigid motion correction was applied to register the individual TI images before inline T1 fitting was performed using a monoexponential three-parameter fit. The T2 mapping images were acquired using a T2-prepared single-shot TrueFISP sequence along the same three short-axis planes with T1 mapping images. The parameters were: TR/TE, 2.1/1.1 ms; FA, 70°; bandwidth, 930 Hz/pixel; FOV, 379 × 308 mm^2^; matrix, 192 × 126 pixels; ST, 8 mm; parallel acquisition acceleration factor, 2, with acquisition on every fourth heartbeat; and T2 preparation times, 0, 25, and 55 ms. After automatic in-plane, nonrigid motion correction, T2 pixel maps were generated by fitting pixel intensities onto a 2-parameter monoexponential signal model.

LGE images were obtained 10 min after contrast injection (0.2 mmol/kg gadolinium contrast, meglumine gadoterate [Dotarem], Guerbet). A phase-sensitive inversion recovery-prepared TrueFISP sequence was used to represent the normal myocardium with the inversion time adjusted to null. The LGE images covered the LV in the same short-axis planes with cine or T1 mapping sequences. A fast, low-angle shot sequence with different inversion times (ranging from 150 to 650 ms to null) was used to determine the inversion time before LGE imaging. Post-contrast T1 mapping images were acquired 15 min after contrast injection along the same three short-axis of LV images used for native T1 mapping with a scheme “4(1)3(1)2” using three inversion pulses. Hematocrit levels were evaluated immediately before cardiac MRI.

### 5.6 CMR analysis

CMR data were analyzed using CVI image analysis software (Circle Cardiovascular Imaging Inc., Calgary, AB, Canada). An experienced radiologist (H. Y. J.) blinded to the patient’s clinical history analyzed the CMR data. Biventricular function was assessed using the end-systolic and end-diastolic volumes from short-axis CINE images by manually delineating the edges of both ventricles' endocardial and epicardial borders. Systolic and end-diastolic biventricular volumes, ventricular mass, stroke volume (SV), and left and right ventricular EF were automatically calculated.

Global native T1, T2, postcontrast T1, and ECV values were measured in 16 myocardial segments. The myocardial borders were excluded with a 10 % offset to reduce partial volume artifacts. ECV values were calculated using the following formula:

ECV (%) = (ΔR1 of myocardium/ΔR1 of left ventricular blood pool) × (1 - hematocrit) × 100.

The native T1 value of the left ventricular blood pool was measured using a circular region of interest of at least 10 mm^2^ to avoid the papillary muscles. Quantitative LGE was measured using the 5SD method [28]. The reference values for native T1, ECV, and T2, obtained using the same cardiac MRI protocol, were 1219.0 ± 29.1 ms, 25.7 ± 2.4 %, and 39.6 ± 2.0 ms, respectively.

### 5.7 Statistical analysis

The study participants were classified into non-CHIP (no defective CHIP-related mutations) and CHIP (at least one CHIP-related mutation in any of the sequenced genes). The presence of CHIP-related mutations was defined as variants with VAF ≥1.5 %.

Continuous variables were expressed as mean ± standard deviation or median (interquartile range [IQR]), while categorical variables were expressed as n (%). Baseline characteristics and cardiac MRI findings were compared according to CHIP mutations using the *t*-test or Mann–Whitney *U* test for continuous variables and the chi-squared test or Fisher’s exact for categorical variables, adjusting for multiple testing with the Benjamini–Hochberg method. The adjusted p-values represent the false discovery rate and p-values <0.05 were considered significant. As part of the sensitivity analysis, a 1:2 age- and sex-matched analysis between the CHIP and non-CHIP groups was performed, defining CHIP-related mutations as VAF ≥2 %. Multivariate regression models were employed to examine the relationship between CHIP and cardiac MRI parameters, adjusting for age, sex, and left ventricular ejection fraction. Additionally, logistic regression was used to assess the prevalence of CHIP with patient diagnosed non-ischemic HFrEF compared to general population, expressed in odds ratio (ORs) with 95 % confidence interval (CIs), and multivariate model was constructed after adjusting for age and sex.

Statistical analyses were conducted using R software, version 4.3.2 (R Foundation for Statistical Computing, Vienna, Austria), and all tests were two-sided with the assumption of *p* < 0.05 being statistically significant.

## CRediT authorship contribution statement

**Jooyeon Lee:** Writing – review & editing, Writing – original draft, Visualization, Software, Investigation, Formal analysis, Data curation. **Yoo Jin Hong:** Writing – review & editing, Writing – original draft, Resources, Methodology, Investigation, Formal analysis, Data curation. **Jin-Ho Park:** Resources, Investigation, Data curation. **Su-Yeon Choi:** Resources, Investigation, Data curation. **Chansub Lee:** Resources, Investigation, Data curation. **Choonghyun Sun:** Resources, Investigation, Data curation. **Hongyul An:** Resources, Investigation, Data curation. **Youngil Koh:** Resources, Investigation, Data curation. **Se-Eun Kim:** Resources, Methodology, Investigation, Data curation. **Jaewon Oh:** Resources, Methodology, Investigation, Data curation. **Seok-Min Kang:** Writing – review & editing, Supervision, Project administration, Investigation. **Chan Joo Lee:** Writing – review & editing, Writing – original draft, Visualization, Validation, Supervision, Software, Project administration, Methodology, Investigation, Funding acquisition, Data curation, Conceptualization. **Young-Jin Kim:** Writing – review & editing, Supervision, Investigation, Data curation, Conceptualization.

## Data and code availability statement

The datasets generated during and/or analyzed during the current study are available from the corresponding author on reasonable request.

## Declaration of competing interest

The authors declare the following financial interests/personal relationships which may be considered as potential competing interests:C.J.L. reports equipment, drugs, or supplies was provided by Genome Opinion Inc. C.J.L reports a relationship with Genome Opinion Inc that includes: funding grants. J.H.P reports a relationship with Genome Opinion Inc that includes: funding grants. S.-Y.C. reports a relationship with Genome Opinion Inc that includes: funding grants. C.L., C.S.,H.A and Y.K reports a relationship with Genome Opinion Inc that includes: employment and equity or stocks. If there are other authors, they declare that they have no known competing financial interests or personal relationships that could have appeared to influence the work reported in this paper.
