# Supplementary material for: Association of clonal hematopoiesis of indeterminate potential with myocardial characteristic differences in non-ischemic heart failure
Source: Heliyon. 2025 Feb 20;11(4):e42858. doi: 10.1016/j.heliyon.2025.e42858 (PMC11904585; doi:10.1016/j.heliyon.2025.e42858)
Supplement: Multimedia component 1 [file mmc1.docx]

**Supplementary Table 1.** Prevalence of CHIP mutations

|  | GENIE Cohort (N=5486) | HFrEF  (N=95) | *p*-value |
| --- | --- | --- | --- |
| *Demographics* |  |  |  |
| Age, years | 55.1±9.4 | 59.0±14.0 | **0.009** |
| Male, n (%) | 3874 (70.6%) | 63 (66.3%) | 0.425 |
| BMI, kg/m^2^ | 24.0±2.9 | 25.2±4.1 | **0.005** |
| *VAF Criterion for CHIP* |  |  |  |
| **VAF** **≥1.5%**, n (%) |  |  |  |
| Any mutations | 661 (12.0%) | 15 (15.8%) | 0.266 |
| DTAP | 448 (8.2%) | 11 (11.6%) | 0.254 |
| DTA | 439 (8.0%) | 11 (11.6%) | 0.186 |
| DTP | 402 (7.3%) | 11 (11.6%) | 0.115 |
| DT | 393 (7.2%) | 11 (11.6%) | 0.108 |
| *DNMT3A* | 313 (5.7%) | 8 (8.4%) | 0.261 |
| *TET2* | 90 (1.6%) | 5 (5.3%) | **0.022** |
| *ASXL1* | 58 (1.1%) | 1 (1.1%) | 1.000 |
| *PPM1D* | 11 (0.2%) | 0 (0.0%) | 1.000 |
| **VAF ≥2.0%**, n (%) |  |  |  |
| Any mutations | 528 (9.6%) | 11 (11.6%) | 0.484 |
| DTAP | 362 (6.6%) | 9 (9.5%) | 0.293 |
| DTA | 357 (6.5%) | 9 (9.5%) | 0.290 |
| DTP | 320 (5.8%) | 9 (9.5%) | 0.180 |
| DT | 315 (5.7%) | 9 (9.5%) | 0.121 |
| *DNMT3A* | 253 (4.6%) | 7 (7.4%) | 0.212 |
| *TET2* | 70 (1.3%) | 4 (4.2%) | 0.037 |
| *ASXL1* | 52 (0.9%) | 1 (1.1%) | 0.599 |
| *PPM1D* | 6 (0.1%) | 0 (0.0%) | 1.000 |

GENIE cohort, cohort study of individuals who underwent a routine health checkup; HFrEF, non-ischemic heart failure patients with ejection fraction≤40%; VAF, variant allele frequency.

DTAP, any mutations of *DNMT3A*, *TET2*, *ASXL1* or *PPM1D*; DTA, any mutations of *DNMT3A*, *TET2* or *ASXL1*; DTP, any mutations of *DNMT3A*, *TET2* or *PPM1D*; DT, any mutations of *DNMT3A* or *TET2*

**Supplementary Table 2.** Logistic regression analyses for prevalence of CHIP mutations in non-ischemic HFrEF adjusted for age and sex

|  | Odds ratio | 95% CI | P value |
| --- | --- | --- | --- |
| **VAF ≥1.5%** |  |  |  |
| Any mutations | 1.06 | 0.60-1.89 | 0.836 |
| DTAP | 1.21 | 0.64-2.30 | 0.560 |
| DTA | 1.25 | 0.66-2.37 | 0.501 |
| DT | 1.37 | 0.72-2.60 | 0.344 |
| DTP | 1.41 | 0.74-2.68 | 0.296 |
| *DNMT3A* | 1.29 | 0.62-2.69 | 0.502 |
| *TET2* | 2.58 | 1.01-6.57 | 0.047 |
| *ASXL1* | 0.77 | 0.11-5.67 | 0.801 |
| *PPM1D* | 0.00 | 0-infinite | 0.978 |
| **VAF ≥2.0%** |  |  |  |
| Any mutations | 0.94 | 0.49-1.80 | 0.848 |
| DTAP | 1.21 | 0.60-2.44 | 0.599 |
| DTA | 1.24 | 0.61-2.49 | 0.556 |
| DT | 1.38 | 0.68-2.78 | 0.375 |
| DTP | 1.41 | 0.70-2.85 | 0.339 |
| *DNMT3A* | 1.38 | 0.63-3.03 | 0.423 |
| *TET2* | 2.59 | 0.91-7.33 | 0.073 |
| *ASXL1* | 0.88 | 0.12-6.43 | 0.895 |
| *PPM1D* | 0.00 | 0-infinite | 0.984 |

HFrEF, non-ischemic heart failure patients with ejection fraction≤40%; VAF, variant allele frequency.

DTAP, any mutations of *DNMT3A*, *TET2*, *ASXL1* or *PPM1D*; DTA, any mutations of *DNMT3A*, *TET2* or *ASXL1*; DTP, any mutations of *DNMT3A*, *TET2* or *PPM1D*; DT, any mutations of *DNMT3A* or *TET2*

**Supplementary Table 3.** Baseline characteristics in age, sex-matched subjects by CHIP mutations

|  | CHIP^a^  (n=15) | Non-CHIP  (n=30) | *p*-value |
| --- | --- | --- | --- |
| *Demographics* |  |  |  |
| Age, years | 67.6±9.6 | 67.1±8.8 | 0.862 |
| Male, n (%) | 10 (66.7%) | 14 (46.7%) | 0.342 |
| *Laboratory findings* |  |  |  |
| Hb, g/dL | 12.8 [12.2;14.0] | 13.9 [12.1;14.3] | 0.289 |
| NT-proBNP, pg/mL | 1105.0 [271.0;1695.0] | 417.0 [158.0;865.5] | 0.179 |
| *Echocardiographic parameters* |  |  |  |
| EF, % | 26.7±8.6 | 31.0±8.2 | 0.110 |
| LVEDD, mm | 62.5±10.2 | 60.8±8.4 | 0.539 |
| LVESD, mm | 54.0±10.4 | 51.6±8.7 | 0.423 |
| *Cardiac medications,* n (%) |  |  |  |
| ARB | 4 (26.7%) | 6 (20.0%) | 0.899 |
| ARNI | 9 (60.0%) | 23 (76.7%) | 0.416 |
| Beta blockers | 12 (80.0%) | 25 (83.3%) | 1.000 |
| MRA | 13 (86.7%) | 27 (90.0%) | 1.000 |
| SGLT2 inhibitors | 3 (20.0%) | 6 (20.0%) | 1.000 |

^a^CHIP mutations with variant allelic fraction (VAF) ≥1.5%.

NT-proBNP , N-terminal pro b-type natriuretic peptide ; EF , ejection fraction ; LVEDD , left ventricular end-diastolic diameter; LVESD , left ventricular end-systolic diameter ; ARB , angiotensin-receptor blocker ; ARNI , angiotensin receptor-neprilysin inhibitor ; MRA , mineralocorticoid receptor antagonist ; SGLT2 inhibitor , sodium-glucose cotransporter type 2 inhibitor

**Supplementary Table 4.** Heart failure medications at outpatient clinic

|  | CHIP^a^  (n=15) | Non-CHIP  (n=80) | *p*-value |
| --- | --- | --- | --- |
| *Cardiac medications,* n (%) |  |  |  |
| ARB | 4 (26.7%) | 18 (22.5%) | 0.986 |
| ARNI | 9 (60.0%) | 62 (78.5%) | 0.231 |
| Beta blockers | 12 (80.0%) | 69 (86.2%) | 0.818 |
| MRA | 13 (86.7%) | 72 (90.0%) | 1.000 |
| SGLT2 inhibitors | 3 (20.0%) | 16 (20.0%) | 1.000 |
| Ivabradine | 2 (13.3%) | 9 (11.2%) | 1.000 |
| NOACs | 4 (26.7%) | 23 (29.1%) | 1.000 |
| Statins | 5 (33.3%) | 45 (56.2%) | 0.177 |

^a^CHIP mutations with variant allelic fraction (VAF) ≥1.5%.

ARB , angiotensin-receptor blocker ; ARNI , angiotensin receptor-neprilysin inhibitor ; MRA , mineralocorticoid receptor antagonist ; SGLT2 inhibitor , sodium-glucose cotransporter type 2 inhibitor ; NOACs , non-vitamin K oral anticoagulants

**Supplementary Table 5.** Baseline characteristics of patients by CHIP mutations with VAF ≥2.0%.

|  | CHIP^b^  (n=11) | Non-CHIP  (n=84) | *p*-value |
| --- | --- | --- | --- |
| ***Demographics*** |  |  |  |
| Age, years | 69.0±10.7 | 57.7±14.0 | **0.012** |
| Male, n (%) | 8 (72.7%) | 55 (65.5%) | 0.889 |
| Clinical risk factor, n (%) |  |  |  |
| HTN | 7 (63.6%) | 31 (36.9%) | 0.169 |
| DM | 5 (45.5%) | 20 (23.8%) | 0.242 |
| CAOD | 1 (9.1%) | 5 (6.0%) | 1.000 |
| Stroke | 1 (9.1%) | 3 (3.6%) | 0.953 |
| SBP, mmHg | 131.1±28.5 | 127.3±21.2 | 0.596 |
| DBP, mmHg | 80.9±19.3 | 84.2±17.1 | 0.56 |
| HR, bpm | 91.2±27.6 | 88.3±24.6 | 0.721 |
| Heart rhythm, n (%) |  |  | 0.460 |
| Atrial fibrillation | 1 (9.1%) | 22 (26.2%) |  |
| Atrial flutter | 1 (9.1%) | 6 (7.1%) |  |
| Sinus | 9 (81.8%) | 56 (66.7%) |  |
| LBBB, n (%) | 1 (9.1%) | 15 (17.9%) | 0.763 |
|  |  |  |  |
| ***Laboratory findings*** |  |  |  |
| Hb, g/dL | 12.8 [12.2;13.4] | 13.9 [12.9;14.9] | **0.065** |
| Total protein, g/dL | 6.9±0.7 | 7.2±0.5 | 0.164 |
| Albumin, g/dL | 4.2±0.6 | 4.5±0.3 | 0.164 |
| Glucose, mg/dL | 110.2±20.4 | 113.6±35.8 | 0.784 |
| Total cholesterol, mg/dL | 144.5±36.8 | 156.8±39.8 | 0.412 |
| Triglyceride, mg/dL | 100.2±33.4 | 135.9±90.4 | 0.386 |
| HDL-cholesterol, mg/dL | 45.0±7.8 | 46.7±10.8 | 0.734 |
| LDL-cholesterol, mg/dL | 71.7±16.1 | 96.0±40.4 | 0.317 |
| BUN, mg/dL | 23.1±10.2 | 19.7±7.4 | 0.180 |
| Cr, mg/dL | 2.0±2.7 | 1.0±0.3 | 0.218 |
| Na, mmol/L | 138.5±3.8 | 139.2±2.7 | 0.458 |
| K, mmol/L | 4.4±0.4 | 4.5±0.5 | 0.732 |
| NT-proBNP, pg/mL | 428.0 [271.0;1695.0] | 179.5 [94.0;682.0] | **0.065** |

^b^CHIP mutations with variant allelic fraction (VAF) ≥2.0%.

HTN , hypertension ; DM , diabetes mellitus ; CAOD , coronary artery occlusive disease ; LBBB , left bundle branch block; SBP , systolic blood pressure; DBP , diastolic blood pressure; HR , heart rate ; Hb , hemoglobin; HDL-cholesterol , high-density lipoprotein-cholesterol ; LDL-cholesterol , low-density lipoprotein-cholesterol ; NT-proBNP , N-terminal pro b-type natriuretic peptide

**Supplementary Table 6.** Cardiac MRI findings according to CHIP mutation with VAF ≥2.0%.

|  | **Total population** | | |  | | **Age, sex-matched** | | |
| --- | --- | --- | --- | --- | --- | --- | --- | --- |
|  | CHIP^b^  (n=11) | Non-CHIP  (n=84) | *p*-value | |  | CHIP^b^  (n=11) | Non-CHIP  (n=22) | *p*-value |
| LVEDV, ml | 237.4±81.7 | 234.0±79.9 | 0.894 | |  | 237.4±81.7 | 211.2±78.6 | 0.380 |
| LVESV, ml | 176.7±74.7 | 174.5±76.8 | 0.929 | |  | 176.7±74.7 | 159.5±73.9 | 0.535 |
| LVSV, ml | 60.7±21.5 | 59.5±20.8 | 0.851 | |  | 60.7±21.5 | 51.7±18.5 | 0.222 |
| LVEF, % | 28.1±12.1 | 27.3±9.9 | 0.800 | |  | 28.1±12.1 | 26.2±8.7 | 0.615 |
| LVCO, l/min | 4.4±1.3 | 4.3±1.5 | 0.970 | |  | 4.4±1.3 | 3.5±1.1 | 0.048 |
| LVCI, l/min/m^2^ | 2.6±0.8 | 2.4±0.7 | 0.392 | |  | 2.6±0.8 | 2.1±0.6 | 0.073 |
| RVEDV, ml | 151.7±40.2 | 176.6±66.8 | 0.231 | |  | 151.7±40.2 | 139.9±53.2 | 0.524 |
| RVESV, ml | 94.0±39.1 | 118.3±61.3 | 0.204 | |  | 94.0±39.1 | 90.2±45.8 | 0.817 |
| RVSV, ml | 57.6±18.1 | 58.3±20.7 | 0.926 | |  | 57.6±18.1 | 49.7±18.5 | 0.249 |
| RVEF, % | 39.4±12.6 | 35.6±12.6 | 0.347 | |  | 39.4±12.6 | 37.7±12.7 | 0.709 |
| RVCO, l/min | 4.2±1.1 | 4.3±1.5 | 0.813 | |  | 4.2±1.1 | 3.3±1.1 | 0.058 |
| RVCI, l/min/m^2^ | 2.5±0.7 | 2.4±0.7 | 0.590 | |  | 2.5±0.7 | 2.1±0.6 | 0.081 |
| Native T1, ms | 1355.2±46.6 | 1314.5±51.7 | **0.015** | |  | 1355.2±46.6 | 1333.9±56.8 | 0.292 |
| T2, ms | 43.3±2.9 | 41.5±3.1 | 0.068 | |  | 43.3±2.9 | 43.6±3.5 | 0.799 |
| ECV, % | 34.0 [32.0;36.7] | 29.9 [27.3;34.4] | **0.010** | |  | 34.0 [32.0;36.7] | 33.0 [30.0;36.8] | 0.330 |
| LGE quantification, % | 18.9 [12.4;25.3] | 8.8 [5.8;12.6] | **0.002** | |  | 18.9 [12.4;25.3] | 9.8 [6.9;12.4] | **0.004** |

^b^CHIP mutations with variant allelic fraction (VAF) ≥2.0%.

CI , cardiac index; CO, cardiac output; ECV, extracellular volume; EDV, end-diastolic volume; EF, ejection fraction; ESV, end-systolic volume; LGE, late gadolinium enhancement; LV, left ventricle; RV, right ventricle.

**Supplementary Table 7.** Multivariate regression model of factors associated with MRI mapping and CHIP mutations with VAF ≥2.0%.

(A) Total population

|  |  | Native T1 | |  | T2 | |  | ECV | |  | LGE | |
| --- | --- | --- | --- | --- | --- | --- | --- | --- | --- | --- | --- | --- |
|  |  | *β* (SE) | *p*-value |  | *β* (SE) | *p*-value |  | *β* (SE) | *p*-value |  | *β* (SE) | *p*-value |
| Age |  | 0.17 (0.39) | 0.666 |  | 0.07 (0.02) | **0.001** |  | 0.05 (0.03) | 0.123 |  | 0.01 (0.06) | 0.900 |
| Male |  | -25.64 (11.14) | **0.024** |  | -0.46 (0.64) | 0.474 |  | -0.98 (0.98) | 0.321 |  | 0.99 (1.64) | 0.547 |
| EF |  | -1.00 (0.67) | 0.142 |  | -0.04 (0.04) | 0.335 |  | -0.01 (0.06) | 0.818 |  | 0.12 (0.10) | 0.223 |
| CHIP^b^ |  | -40.42 (16.73) | **0.018** |  | -1.00 (0.97) | 0.307 |  | -3.10 (1.48) | **0.039** |  | -8.75 (2.46) | **0.001** |

(B) In age, sex-matched subjects by CHIP mutations

|  |  | Native T1 | |  | T2 | |  | ECV | |  | LGE | |
| --- | --- | --- | --- | --- | --- | --- | --- | --- | --- | --- | --- | --- |
|  |  | *β* (SE) | *p*-value |  | *β* (SE) | *p*-value |  | *β* (SE) | *p*-value |  | *β* (SE) | *p*-value |
| Age |  | 0.63 (1.06) | 0.554 |  | 0.05 (0.07) | 0.496 |  | 0.03 (0.09) | 0.755 |  | -0.04 (0.16) | 0.813 |
| Male |  | -47.0 (19.9) | **0.026** |  | 0.63 (1.33) | 0.639 |  | -0.49 (1.64) | 0.769 |  | -0.55 (3.03) | 0.858 |
| EF |  | -0.21 (1.07) | 0.845 |  | -0.05 (0.07) | 0.471 |  | -0.00 (0.09) | 0.968 |  | 0.02 (0.16) | 0.890 |
| CHIP^b^ |  | 34.31 (19.84) | 0.095 |  | -0.51 (1.32) | 0.705 |  | 1.40 (1.63) | 0.398 |  | 8.97 (3.02) | **0.006** |

^b^CHIP mutations with variant allelic fraction (VAF) ≥2.0%.

CHIP , Clonal Hematopoiesis of Indeterminate Potential; ECV, extracellular volume; EF, ejection fraction ; LGE, late gadolinium enhancement; SE, standard error

**Supplementary Figure 1.** Gene variants in nonischemic HF patients with CHIP mutations according to variant allelic fraction


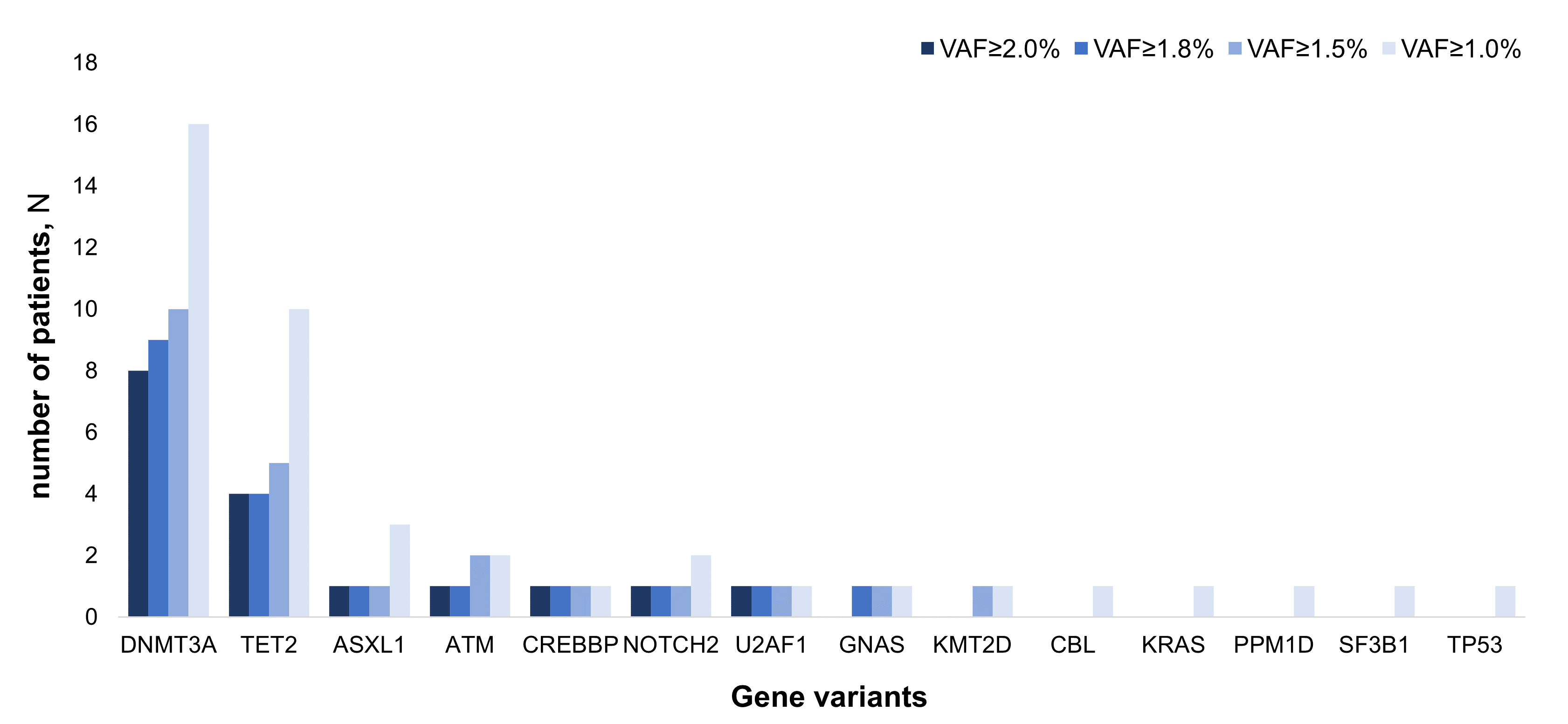


VAF, variant allelic fraction

**Supplementary Figure 2.** Schematic diagram of the study flow


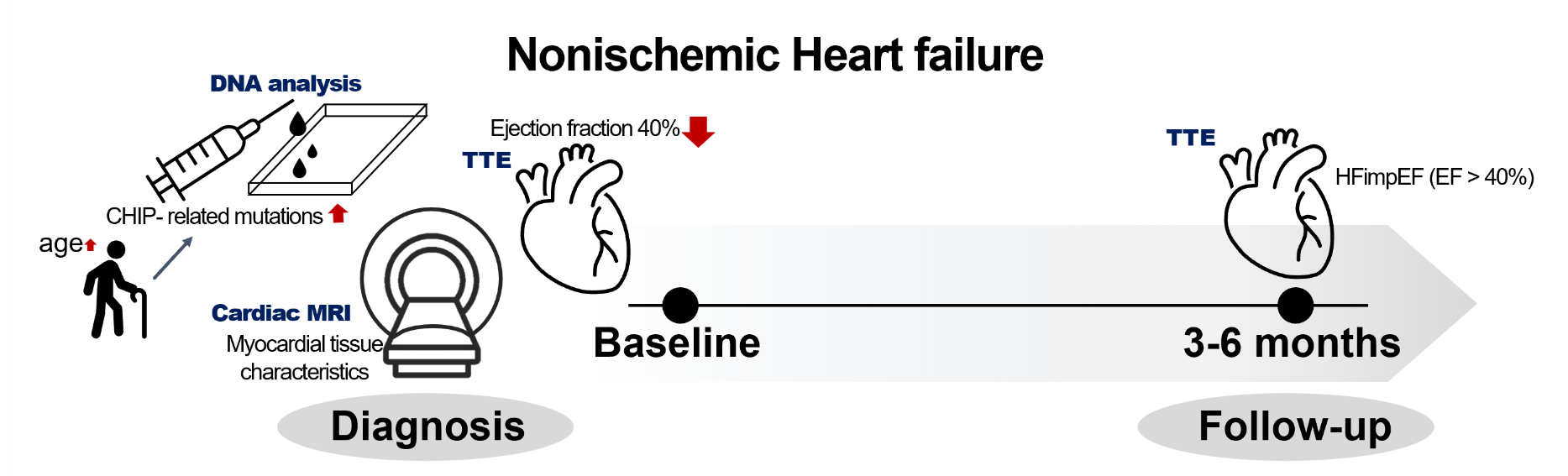


CHIP, Clonal Hematopoiesis of Indeterminate Potential ; HFimpEF, heart failure with improved ejection fraction ; MRI, magnetic resonance imaging ; TTE, transthoracic echocardiography.
